# Supplementary material for: A UMLS-based spell checker for natural language processing in vaccine safety
Source: BMC Med Inform Decis Mak. 2007 Feb 12;7:3. doi: 10.1186/1472-6947-7-3 (PMC1805499; doi:10.1186/1472-6947-7-3)
Supplement: Additional file 13 — RAP application directory. Sets up directory for RAP (RDF application for PHP) source code [file 1472-6947-7-3-S13.gz › rap/doc/tests/Test_Set_Database_Connection.htm]

Test Set Database Connection


### RDF API for PHP V0.9.1

# Test: Set Database Connection

This example is part of the RAP - Rdf API for PHP documentation.

|  |  |  |
| --- | --- | --- |
| **Note:** |  | RAP uses ADOdb Library for PHP V4.52 (http://adodb.sourceforge.net/), which allows you to connect to multiple databases in a portable manner. For a list of supported databases see the ADOdb documentation. |

  
  

### 1. Connect to MsAccess database (via ODBC) and create tables.

Set the system DSN for the particular database file (control pannel -> administrative
tools -> data sources (odbc) -> system DSN).

`// Include RAP  
define("RDFAPI_INCLUDE_DIR", "./../api/");  
include(RDFAPI_INCLUDE_DIR . "RDFAPI.php");  
   
// Connect to MsAccess database (rdf_db DSN) using connection settings  
// defined in constants.php :  
// -------------------------------------------------------  
// Database  
// -------------------------------------------------------  
// define("ADODB_DB_DRIVER", "odbc");  
// define("ADODB_DB_HOST", "rdf_db");  
// define("ADODB_DB_NAME", "");  
// define("ADODB_DB_USER", "");  
// define("ADODB_DB_PASSWORD", "");  
$rdf_database = ModelFactory::getDbStore();  
   
// Create tables for MsAccess  
$rdf_database->createTables('MsAccess');`

  
  

### 2. Connect to MySQL database and create tables.

`// Connect to MySQL database with user defined connection settings  
$rdf_database = ModelFactory::getDbStore('MySQL', 'host', 'db_name', 'user_name', 'password');  
   
// Create tables for MySQL  
$mysql_database->createTables('MySQL');`

  
  

### 3. Connect to other databases

`// Example:  
// Connect to Oracle database with user defined connection settings  
$oracle_database = ModelFactory::getDbStore('Oracle', FALSE, 'db_name', 'username', 'password' );`

  

|  |  |  |
| --- | --- | --- |
| **Note:** |  | Method createTables() currently supports only MySQL, MS SQL Server, and MsAccess.  If you want to use other databases, you will have to create tables by yourself according to the abstract database schema provided with the API documentation. |

  
  

### 4. Close the database connection

`// Close the connection   
$rdf_database->close();`

  

|  |  |  |
| --- | --- | --- |
| **Warning:** |  | If you close the rdf database all active instances of DbModel from this DbStore will lose their database connection !!! |
